# National shortage of home intravenous inotrope agents: The “inotrope pandemic” continues

**DOI:** 10.1016/j.jhlto.2023.100023

**Published:** 2023-11-19

**Authors:** Dipan Uppal, Ersilia M. DeFilippis, Jillian Dietz, David Baran, Jerry D. Estep, Mauricio Velez, Nina Thakkar Rivera, David Snipelisky

**Affiliations:** a Department of Cardiology, Section of Heart Failure & Cardiac Transplant Medicine, Cleveland Clinic Florida, Weston, Florida; b Center for Advanced Cardiac Care, Division of Cardiology, Columbia University Irving Medical Center, New York, New York

**Keywords:** inotropes, national shortage, heart transplantation, end-stage heart failure, palliative care, reimbursement

The use of intravenous inotropic medications has increased substantially as the epidemiology of heart failure continues to shift in the direction of more patients with advanced disease.^1^ Although inotropic support, particularly in the outpatient setting, has historically been reserved for symptom improvement and palliative measures, use as a bridge to heart transplantation (HT) continues to rise.^2^, ^3^, ^4^ In 2016, the 21st Century Cures Act was passed with a focus on increased funding for biomedical research, yet more controversial changes affected reimbursement methodology for all home infusion medications, including inotropes. An initial call to action highlighted the lack of appropriate reimbursement structures and raised concerns surrounding obtaining intravenous inotropes in the home setting.^5^ Several years have passed with recent reports of drug shortages and difficulty in coordination of care outside of the hospital. Therefore, we sought to assess whether transplant programs were experiencing difficulties in home inotrope coordination in the current era and the impact on HT candidates and their management.

Institutional Review Board approval was obtained and an online survey was sent to providers at HT programs throughout the United States. Of 147 active programs, 53 (36%) completed the survey (Table 1). The majority were academic teaching hospitals (*n* = 24, 45%) and had over 500 beds (*n* = 40, 76%) with wide geographical representation. Almost all programs were aware of the current inotrope shortage (*n* = 50, 94%) and the majority (*n* = 36, 68%) reported an impact on current patient management.

**Table 1:** Survey Responses

Adult heart transplant centers surveyed (*n* = 53)	
*Hospital affiliation* Community-based teaching hospital: 9 (17%) Community-based nonteaching hospital: 7 (13%) Academic teaching hospital: 24 (45%) University-based hospital: 13 (25%)	
*Hospital size* Less than 250 beds: 2 (4%) 250-500 beds: 11 (21%) Greater than 500 beds: 40 (76%)	
*Geography* Northeast: 10 (19%) Southwest: 5 (9%) West: 8 (15%) Southeast: 14 (26%) Midwest: 16 (30%)	
*Transplant volume (2022)* 1-10: 13 (25%) 11-30: 16 (30%) 31-50: 12 (23%) >50: 12 (23%)	
*LVAD volume (2022)* 0-10: 14 (26%) 11-30: 21 (40%) 31-50: 14 (26%) >50: 4 (8%)	
Are you aware of the national shortage and/or difficulty obtaining IV inotropes for patients at home?	Yes: 50 (94%) No: 3 (6%)
Centers currently experiencing difficulties in obtaining home inotropes (*n* = 36, 68%)	Centers not currently experiencing difficulties in obtaining home inotropes (*n* = 17, 32%)
*Extent of impact* Somewhat: 11 (31%) Moderate: 17 (47%) Significant: 8 (22%)	*Anticipate future impact on your practice* Yes: 11 (65%) No: 6 (35%)
*Impacted decision to initiate patients on inotropes in the hospital* Yes: 19 (53%) No: 17 (47%)	*Extent of future impact* Somewhat: 7 (64%) Moderate: 4 (36%)
*Hospital length of stay* Decreased: 1 (3%) Stayed the same: 7 (19%) Increased: 28 (78%)	*Anticipate being less likely to initiate inotropes in the hospital with the goal of discharge home* Yes: 4 (36%) No: 7 (64%)
*Less likely to list a patient for transplant with the goal of discharge on home inotrope* Yes: 14 (39%) No: 22 (61%)	*Anticipate an increase in hospital length of stay* Yes: 9 (82%) No: 2 (18%)
*More likely to keep a patient admitted in the hospital while actively listed for heart transplant* Yes: 20 (56%) No: 16 (44%)	*Anticipate being more aggressive in weaning inotropes in the hospital* Yes: 5 (45%) No: 6 (55%)
*More aggressive in weaning inotropes with goal to discharge home without inotrope* Yes: 21 (58%) No: 15 42%)	*Anticipate being more likely to keep a patient in the hospital while actively listed for transplant* Yes: 3 (27%) No: 8 (73%)
*More likely to use alternative support strategies for transplant listing, including temporary mechanical circulatory support such as intra-aortic balloon pumps or Impella devices* Yes: 19 (53%) No: 17 (47%)	*Anticipate being more likely to use alternate support strategies for transplant listing, including temporary mechanical circulatory support such as intra-aortic balloon pumps or Impella devices* Yes: 4 (36%) No: 7 (64%)
*More likely to consider durable LVAD therapy as bridge to transplant strategy for inotrope-dependent patients* Yes: 21 (58%) No: 15 (42%)	*More likely to consider durable LVAD therapy as a bridge to transplant strategy for inotrope-dependent patients* Yes: 4 (36%) No: 7 (64%)

Abbreviation: LVAD, left ventricular assist device.

Of the hospitals reporting an impact, the vast majority described the impact as being either a moderate or significant impact (*n* = 25, 69%) with 53% endorsing a change in practice on the decision of whether inotropes are initiated in the hospital setting. Seventy-eight percent (*n* = 28) reported increased hospital length of stay and over half stated they were more likely to keep a patient admitted to the hospital while actively listed for HT rather than discharge home on inotropes. More than half of the respondents were more likely to consider durable left ventricular assist device (LVAD) therapy as a bridge to transplant among inotrope-dependent patients because of the shortage.

Of the respondents not currently experiencing issues (*n* = 17, 32%) with obtaining home inotropes, 65% (*n* = 11) anticipate experiencing difficulties accessing home inotrope therapy in the future with almost all anticipating significant increases in length of stay. Respondents not currently experiencing issues with home inotrope coordination are more likely to use inotropes in the hospital yet do anticipate utilizing other temporary mechanical circulatory support options, such as intra-aortic balloon pumps and Impella devices, as bridges to transplant and are also more likely to utilize durable LVAD therapy as a bridge to transplant.

Our data highlight the challenges associated with the 21st Century Cures Act that continue to plague our patients with advanced HF.^5^ Our study assessed a population not otherwise evaluated in the literature and demonstrates a growing problem among patients evaluated and listed for HT. The majority of programs surveyed are experiencing difficulties in coordinating home inotropes with a change in management strategies because of this. Furthermore, of those programs not actively experiencing difficulties in coordinating home inotropes, most anticipate issues in the future.

Although a separate provision of the 21st Century Cures Act did allow for an increase in reimbursement in 2021 following its initial inception in 2016, reimbursement issues still persist and the inotrope pandemic continues with no concrete end in sight. Medication shortages have evolved as a result of COVID-19, yet the difficulty in obtaining inotropes persists despite the resolution of the medication shortage, therefore we suspect that much of the on-going issues result from the lack of reimbursement. The paradoxical decrease in number of available home inotropes has led to an increase in overall health care spending, particularly as a result of recurrent hospitalization and increased patient length of stay, as well as a shift in patient management with a focus on other mechanical support devices and strategies for transplant listing.^5^ Our data provide a more contemporary call to action to improve reimbursement methodology strategies as the inotrope pandemic evolves.

## Disclosure statement

The authors declare that they have no known competing financial interests or personal relationships that could have appeared to influence the work reported in this paper.
